# Alcoholic Beverages Drinking among Female Students in a Tourist Province, Thailand

**DOI:** 10.5539/gjhs.v4n1p57

**Published:** 2012-01-01

**Authors:** Wirin Kittipichai, Hatairat Sataporn, Nithat Sirichotiratana, Phitaya Charupoonphol

**Affiliations:** Faculty of Public Health, Mahidol University Bangkok, Thailand E-mail: phwkt@mahidol.ac.th; Anuban Phrasamutchedee School Samutprakarn Province, Thailand E-mail: hatairat_ph9@hotmail.com; Faculty of Public Health, Mahidol University Bangkok, Thailand E-mail: phnsr@mahidol.ac.th; Faculty of Public Health, Mahidol University Bangkok, Thailand E-mail: phpcr@mahidol.ac.th

**Keywords:** Female student, Drinking, Alcoholic beverage, Tourist province

## Abstract

This study aimed to investigate alcoholic beverages drinking and predictive factors among female students. The participants were 377 subjects from 3 high schools in a tourist province, of Thailand. Data collection was done through self-administered questionnaire. Scales of the questionnaire had reliability coefficients ranging from 0.84 – 0.88. The data were analyzed by using descriptive and inferential statistics. The findings revealed as follows. About half (51%) of them have ever drunk and 10.5% of drinkers have drunk once a week. In addition, 15.6% of drinkers began their first drink when they were under 10 years old. Risk factors for alcohol consumption of female student were age, GPA, drinker in family, peer pressure, advertisement and accessibility to alcoholic beverages while protective factors were perception of drinking impacts on family and moral values. Students who have a drinking family member were 4.6 times more likely to drink than those who do not have.

## 1. Introduction

Alcoholic beverage drinking is one of the causes of health problems, crimes, economics, and social problems. Both government and private sectors emphasize politically on alcoholic beverages drinking and participate in activities designed to prevent drinking (Social surveillance and Warning Center, online). Alcohol industry is actively participated in social and school activities, including sport activities, through corporate social responsibility (CSR) program in order to reach out to the target group, who are youth and teenagers. The Thai National Statistical Office in Thailand reported that in 2007, Thai population older than 15 years old consumed alcoholic beverages about 14.9 million people (29.3%), while 15 – 24 years old age group who were drinkers about 2.3 million people (21.9%) (Sornpaisarn, *et al*, 2008) Prevalence rate of lifetime drinking among students aged 10 – 22 years old were 30.5% and 18.2% for boys and girls, respectively (Assnangkornchai, Mukthong, & Intanont, 2009). Between 1999– 2007, it was found that prevalence rate of lifetime drinking among aged 15 – 19 years old increased from 4.7% to 8.0% or 70%. Moreover, girls tend to drink more than boys when comparing increased percentage of drinkers. In addition, parental attitudes toward alcohol and having drinkers in family, contribute to the risk that youth will likely become a drinker. It is estimated that there will be 67% of drinkers in year 2047 (Sornpaisarn, Kaewmungkun, & Wattanaporn, 2010). Consequences of drinking for young women include academic problems, poor health, mental health problems, accidents, as well as the development of alcohol or other drugs dependence (CASA, 2003).

## 2. Objectives of research

The objective of this study was to investigate alcoholic beverages drinking and predictive factors among female students in high schools.

## 3. Methods

### 3.1 Research design and sample

This study was conducted as a cross-sectional survey among female students from 3 high schools in Chonburi province, in eastern Thailand. Chonburi is well-known as an important tourism area on the Gulf of Thailand. Three hundred and thirty-seven female students from grades 1 – 6 were recruited. Data collection was conducted between August and September 2010.

### 3.2 Research instruments

The research instrument was the self-administered questionnaire, consisted of 6 parts: 1) alcoholic beverage drinking, 2) family factors: marital status of parents, drinker in family 3) peer pressure, 4) perception of alcoholic beverage advertisement, 5) accessibility to alcoholic beverages and 6) perception of drinking impacts on health, family, social and moral values. Content validity has been reviewed and approved from 3 experts, one in behavioral science, one in family medicine, and one in health policy. Data were verified with internal consistency reliability coefficients between 0.84 and 0.88. This study was approved by the Ethics Committee for Research in Human Subjects of the Faculty of Public Health, Mahidol University (Ref.No. MUPH 2010-131).

### 3.3 Statistical Analysis

The SPSS for windows was used in both descriptive and inferential statistical analysis of the collected data. The descriptive statistics were frequency, percent, and standard deviation. In addition, Chi-square, t-test, and Multiple Logistic Regression were applied in finding the factors correlating and predicting the alcoholic beverages drinking among female students. Significant level was set to be .05.

## 4. Results

### 4.1 descriptive statistics

The samples were between the age of 12 and 17 years old, with the average age was 15.2. Majority of samples (91%) lived with parents or relatives. Out of 377 female students, 74.5% lived with cohabited parent. More than 9 in 10 (92%) had at least one drinker in the family. Approximately 51% of female students have ever drunk. Female drinkers reported having their first drink between the age of 3 and 17 years old (Mean+S.D.= 12.50+2.32). Approximately 35% of female drinkers had their first alcoholic beverages drinking before high schools, and 15.6% began to drink alcoholic beverages when they were under 10 years old. Regarding the reasons that may encourage their first drinking, more than 56% of female student drinkers said they just wanted to try. Secondly, they started to drink in the party or because of peer pressure (38.1%). Wine or Spy (50.5%) is majority of alcoholic beverages which were chosen, followed by beer (25%) and alcoholic smoothies (15.1%) respectively. Approximately one-third (32.8%) of drinkers drank at home. This study reported that 37.3% of female drinkers drank with their parents or relatives, and more than half (56.2%) drank with their friends. More than 4 in 10 (46%) drank once in a few months, followed by few times a month (44%). One in ten (10%) of female drinkers drank once a week (Table 1).

**Table 1:** Alcohol beverages drinking of female students

Item	Frequency	Percent
**Current drinking (n=377)**		
Drink	192	50.9
Not drink	185	41.1
**Age at first drink (n=192)**		
Under 10 years old	30	15.6
11 – 12 years old	37	19.3
13 – 14 years old	97	50.5
15 – 17 years old	28	14.6
**Reasons for first drink (n=192)**		
Try to drink	108	56.1
Peer pressure	73	38.1
Feel stressed or anxious	9	4.7
Drink followed family members	2	1.1
**Alcoholic beverages at first drink (n=192)**		
Wines or Spy	97	50.5
Beer	48	25
Alcoholic smoothies	29	15.1
Whisky or Vodka or Rice whisky	18	9.4
**Places of drinking (n=192)**		
Home	63	32.8
Dormitory	42	21.9
In party or Restaurant	87	45.3
**Persons who always drinking (n=192)**		
Peers	119	56.2
Parent or Relative	62	37.3
Alone	11	6.5
**Favorite alcoholic beverages (n=192)**		
Wines or Spy or alcohol mixed with fruit-juice	97	50.5
Beer	37	19.3
Alcoholic smoothies	32	16.7
Whisky or Vodka or Rum	26	13.5
**Drinking frequency (n=192)**		
Everyday	1	0.5
Always (3 – 4 times a week)	5	2.6
Sometimes (1 – 2 times a week)	14	7.3
Once a month	84	43.8
Once a few months or less	88	45.8

### 4.2 Inferential statistics

In focusing on the family predictors, namely marital status of parents and drinker in family, the study found that drinking associated with drinker in family with statistical significance at .01. Nonetheless there was no correlation with marital status of parents (Table 2). Additionally, the personal predictors in terms of age and GPA, indicated that drinkers had older age than non-drinkers, whereas the non-drinkers had higher GPA than drinkers with statistical significance at .001 (Table 3).

**Table 2:** Families predictors associated with alcoholic beverages drinking

Family predictors	Drinker	Non-drinker	χ^2^	Odd ratio
*Marital status of parent*				
Cohabitation	135 (48.0)	146 (52.0)	3.677	0.63
Divorce/Separate/Father or mother dead	57 (59.4)	39 (40.6)		
*Drinker in family*				
Yes	186 (53.6)	161 (46.4)	12.641**	4.43
No	6 (20.7)	23 (79.3)		

** p < 0.01

**Table 3:** Means comparison of the scale predictors by alcoholic beverages drinking

Predictors	group	n	Mean	S.D.	t-value
*Personal*					
Age	Drinker	192	15.16	1.076	5.462***
	Non-drinker	185	14.61	0.853	
GPA	Drinker	159	3.01	0.543	3.658***
	Non-drinker	185	14.61	0.853	
*Social*					
Peer pressure	Drinker	189	21.91	7.303	10.945***
	Non-drinker	182	15.40	3.623	
Perceived alcoholic advertisement	Drinker	192	16.12	7.432	8.881***
	Non-drinker	185	10.72	3.881	
Accessibility to alcoholic beverages	Drinker	190	2.75	1.504	5.099***
	Non-drinker	184	1.98	1.433	
*Perception of drinking impact on*					
Health	Drinker	191	23.30	41.69	5.986***
	Non-drinker	185	25.72	3.665	
Family	Drinker	192	19.85	5.682	5.026***
	Non-drinker	185	22.36	3.868	
Social value	Drinker	191	16.84	3.891	3.343***
	Non-drinker	184	18.03	2.952	
Moral value	Drinker	191	11.50	2.294	4.643***
	Non-drinker	185	12.56	2.100	

*** p < 0.001

With regard to the social predictors such as peer pressure, perception of alcoholic beverages advertisement, and accessibility to alcoholic beverages, comparing the perception scores by alcoholic beverages drinking indicated that drinkers had a higher mean score than non-drinkers, with statistical significance at .001. Referring to perception of drinking impact on health, family, social, and moral values, the difference were statistically significant at .001, with drinkers had less mean scores of drinking impacts than non-drinkers (Table 3).

The findings of association between predictors comprised of personal factors, family factors, social factors, and perception of drinking impacts and alcoholic beverages drinking among female students in high schools revealed that there were 10 predictors which were significantly associated with drinking. The significant predictors were age, GPA, drinker in family, peer pressure, advertisement, accessibility of alcoholic beverages, perceptions of drinking impacts on: health, family, social, and moral values. These variables were analyzed to see how it influences alcoholic beverages drinking, by using Multiple Logistic Regression.

The results of analysis (Table 4) revealed that age, drinker in family, peer pressure, advertisement, accessibility to alcoholic beverages, perception of drinking impact on family and moral values, were predictive of alcoholic beverages drinking with statistical significant at .05. Besides the equation was 77.3% correct in predictive of drinkers and 75.5% correct in predictive of non-drinkers, or 76.4% capable of classifying drinkers and non-drinkers. This finding can make clear as follow.

**Table 4:** Multiple logistic regression between predictors and alcoholic beverages drinking

Predictors	B	Odds Ratio	95% CI
Age	0.683***	1.980	1.463 - 2.680
Grade Point Average (GPA)	-0.435	0.647	0.359 - 1.168
Drinker in family^1^	1.529**	4.614	1.438 - 14.802
Peer pressure	0.164***	1.179	1.100 - 1.263
Alcoholic beverages advertisement	0.063*	1.065	1.000 - 1.133
Accessibility to alcoholic beverages	0.242**	1.274	1.066 - 1.523
Perception of drinking impact on			
- Health	-0.007	0.993	0.910 - 1.085
- Family	-0.098*	0.906	0.825 - 0.995
- Social	0.105	1.111	0.970 - 1.272
- Moral values	-0.198**	0.820	0.707 - 0.952
Constant	-10.183		

1 Reference group: no

1) Students live with drinker in family are 4.6 times more likely to drink than those who out drinker in family.
2) For each additional year the students was 1.98 time more likely to be a drinker or 98% [(1.98-1)*100] more likely to be a drinker.
3) Each additional score for accessibility to alcoholic beverages means 1.274 times more likely to be drinker or 27.4% [(1.274-1)*100] more likely to be a drinker.
4) Each additional score for peer pressure means 1.179 times more likely to be drinker or 18% [(1.179-1)*100] more likely to be a drinker.
5) Each additional score for perceived alcohol advertisement means 1.065 times more likely to be drinker or 6.5% [(1.065-1)*100] more likely to be a drinker.
6) Each additional score for perception of drinking impact on moral values means 0.906 times less likely to be drinker or 18% [(1-0.906)*100] less likely to be a drinker.
7) Each additional score for perception of drinking impact on family means 0.820 times less likely to be drinker or 9.4% [(1-0.820)*100] less likely to be a drinker.

Then, alcohol drinking of female students had risk factors such as age, drinker in family, peer pressure, perceived alcohol advertisement, and accessibility to alcoholic beverages, and protective factors, namely perception of drinking impact on family and moral values.

## 5. Discussion

The study indicated 50.9% of female students in high schools had ever drunk alcoholic beverages. This information indicated a higher rate than previous study of Pichainarong & Chaveepojnkamjorn (2010) that showed only 8.8% in central Thailand. Moreover, this study reported a higher percentage of female students drinkers than the study of Assnangkornchai, Mukthong & Intanont (2009), which was a surveyed with high school students in Thailand, between December 2007 and February 2008. It was reported that 18.2% of female students in high schools had ever drunk alcoholic beverages. The high percentage of female student drinkers in current study may be affected by the context of this study. Chonburi is well-known by foreigners as tourist city. Therefore, there are a lot of accessibility to alcoholic beverages such as cocktail lounges, discotheques, nightclubs, convenient stores, and groceries in many areas. These may be encouraging factors for female students to try drinking alcoholic beverages. Environmental factors in social entertainment may induce a person to touch alcohol (Lunsay, 1997). That is, the percent of female students who had experience with drinking in Chonburi is higher than other provinces which are not tourist destinations. It is consistent with a study of Paupongsakorn in 2005, which stated that one of all causes which had effect on alcoholic beverages drinking was easy access to alcoholic beverages, or having stores near home and school.

The results of this study indicated that about 35% of female students had their first drink of alcoholic beverage at the age under 13. It is consistent with the results of school girls surveyed in the United State of America, which indicated that nearly 1 in 4 started to drink for the first time before age 13. It was a higher percentage than the survey in 1960 which indicated only 7% of all girls (CASA, 2006). This is particularly disturbing since those who initiate alcohol drinking in early life are at increased risk of becoming problem drinkers (Grant & Dawson, 1997). It would be more troubling issues for Thai female students which tend to start drinking early in their life. With regard to drinking prevention, previous study found that family supervision and support were important factors to prevent substances used for both girls and boys effectively, especially important for girls (Amaro, Blake, Schwartz, & Flinchbbaugh, 2001). Interestingly, this study stated that 37.3% of female students generally drank alcoholic beverages with their parent or relatives at home. The results are consistent with the previous study of Valentine, Jayne & Gould (2010) who found that parents actually introduce their own children to alcohol at home at an early age. This is especially crucial issue when considering why the parents allow female students who are under their responsibility to drink alcoholic beverages. Drinking is accepted by social as a normal activity or familiarity with alcohol may enhance self-protection for female students. The results in the current research indicate the difference from the norm of Thai traditional society, which parents must be the role model for their child and should not allow the child to drink.

There are many factors that control drinking behavior such as perception of drinking impacts on family and moral values. However, the social problems that may influence the drinking behavior such as easy access to buy alcoholic beverages and peer pressure still exist. Other important factor that may increase the risk for alcohol abuse is parental acceptance of female students’ drinking together with high percent of having family members who drink alcohol (92%). Moreover, the alcoholic beverages advertisement has the indirect effect to the alcohol consumption of female students. The advertised issues have enhanced drinking by making their images as someone with modern life style, attractive person, or hero for the target groups, especially the young. Young people were stimulated to alcohol consumption by advertisement. This study found that 50% of female drinkers have the first drink as self-trial, and 38.1% of them from peer pressure. It is consistent with the study of Boonleu (2003) who studied Thai youth at the age of 13 – 21 which found that alcohol drinking of these participants was influenced by persuasion of friends and self-trial. It is similar with a study of CASA reported in 2003 that alcohol advertising appeals to girls by making drinking as fun and sexy. Experts agreed that advertisement exerts an influence on youth drinking (Grube, 1993). It is consistent with other studies in Thailand such as Kajorndhum, Kajorndhum, & Sornpaisarn (2004) and Sornpaisarn, *et al*. (2008) which reported the related findings.

In conclusion, the significant predictors toward alcoholic beverages drinking of female students would be more of challenging problems to families and policy regulators. This is especially troubling for each family in Thai society, to be concerned about drinking impacts on social, moral values, economics, and well-being both short and long terms, while family structure seems to be fragile. In reality, these families would be responsible for each problem in the future, which can be destroyed or may not rectify chronic social problems.
